# Considering a Non-Constant Anisotropicity Parameter in the Giesekus Model

**DOI:** 10.3390/polym17182510

**Published:** 2025-09-17

**Authors:** Fatemeh Karami, Pavlos S. Stephanou

**Affiliations:** 1 Department of Mechanical Engineering, Lorestan University, Khorramabad 68151-44316, Iran; karami.fa@lu.ac.ir; 2 Department of Chemical Engineering, Cyprus University of Technology, P.O. Box 50329, Limassol 3603, Cyprus

**Keywords:** rheological model, polymer melts, Giesekus model, mobility tensor, friction tensor, Curtiss–Bird model, link tension coefficient

## Abstract

The Giesekus model has proven to be one of the most successful constitutive rheological models. Although Giesekus introduced the anisotropicity parameter as a constant, recent evidence suggests that it should not be. We elaborate herein on the implications of having a variable anisotropicity coefficient; to our knowledge, this is only the second such model. We find that the modification leads to important differences in the predictions of the second normal stress coefficient in simple shear flow, of which the most significant is the shift of the linear viscoelastic envelope of the second normal stress coefficient to higher values in the case of start-up simple shear flow, which is more in line with experimental data in the literature.

## 1. Introduction

The use of plastic in almost every aspect of our everyday lives positions the plastic industry as one of the most important industries. Thus, the optimization of processes producing polymeric materials is of the utmost importance to avoid process instabilities [1,2]. Such a task necessitates the execution of numerical simulations that, at their heart, employ an accurate and reliable constitutive rheological model based on molecular principles [2]. The Giesekus model is widely regarded as one of the most successful constitutive equations for polymer solutions and melts, as it can predict a non-zero second normal stress difference (which is customarily neglected despite evidence to the contrary [3]), stress overshoot during the start-up of simple shear flow, and finite elongational viscosity [4,5,6,7,8]. It has proven accurate in predicting the rheological behavior of lubricants [9], the prediction of secondary flows when viscoelastic fluids flow in channels [3], the flow of drilling mud through a wellbore annulus [10], the flow of a dilute polymer solution in a converging pipe [11], and even the flow of plant-based meat analogs [12]. It introduces the notion that in sufficiently concentrated polymer solutions and melts, the frictional properties should be anisotropic [13,14], controlled by the magnitude of the anisotropicity or Giesekus parameter, *α.* By invoking the phase-space theory for concentrated polymer solutions and melts by Curtiss and Bird [15,16,17], Giesekus [18] showed that his anisotropicity parameter can be related to their link tension coefficient, *ε*, via $\alpha =\left(1-\varepsilon \right)/\left(2+\varepsilon \right)$. Stephanou et al. [19,20] provided the theoretical basis indicating that the link tension coefficient should not be considered a constant but should depend on the nematic order parameter. The revised framework predicts a vanishing *ε* at equilibrium, which remedies the erroneous predictions of the Curtiss–Bird model when considering a constant *ε*, namely, the approach of the transient shear viscosity as *t* → 0 to a constant value and the spurious sign changes of the transient second normal stress coefficient in start-up simple shear flow.

Given the modification proposed by Stephanou et al. [19,20], we herein revisit the original Giesekus model by considering a non-constant anisotropicity parameter following the relationship between *α* and *ε*, and the relation between the former and the nematic order parameter as proposed by Stephanou et al. [19,20]. As such, at equilibrium, the anisotropicity parameter is equal to ½. This is only the second model proposing the notion of a variable anisotropicity parameter, after the molecularly derived, modified Giesekus model proposed by Ilg and Kröger [21]. We elaborate on the implications of the modified Giesekus predictions when considering a variable anisotropicity parameter and discuss whether its predictions are more in line with rheological experimental data. In Section 2, we present the original and modified Giesekus constitutive model and proceed to Section 3, where we compare the modified Giesekus model predictions with the predictions of the original Giesekus, Leonov, and Ilg–Kröger models and against experimental data. We conclude in Section 4 where the most important outcomes of our work are summarized and where we provide our future plans.

## 2. Constitutive Model

We consider an isothermal and incompressible flow. The Giesekus model in this case reads as follows [22]:

(1a) $${\dot{c}}_{\alpha \beta ,[1]}=-\frac{1}{\tau_{R}}\left[\frac{1}{2}\left(c_{\alpha \gamma }\beta_{\beta \gamma }+c_{\beta \gamma }\beta_{\alpha \gamma }\right)-\beta_{\alpha \beta }\right].$$

where **c** is the dimensionless conformation tensor (defined as the average dyadic of the end-to-end vector of polymer chains) and we have defined the upper-convected time derivative (note the use of Einstein’s implicit summation convention for repeated Greek indices):

(1b) $${\dot{c}}_{\alpha \beta ,[1]}\equiv \frac{\partial c_{\alpha \beta }}{\partial t}+u_{\gamma }\nabla_{\gamma }c_{\alpha \beta }-c_{\alpha \gamma }\nabla_{\gamma }u_{\beta }-c_{\gamma \beta }\nabla_{\gamma }u_{\alpha }.$$

For the dimensionless mobility tensor, we consider the linear expression considered by Giesekus [18,22], namely,

(1c) $$\beta_{\alpha \beta }=\delta_{\alpha \beta }+\alpha \left(c_{\alpha \beta }-\delta_{\alpha \beta }\right).$$

Also, the stress tensor is given as,

(2) $$\sigma_{\alpha \beta }=G\left(c_{\alpha \beta }-\delta_{\alpha \beta }\right).$$

In this work, we consider the Giesekus parameter to be given explicitly by an expression relating it to the link tension coefficient:

(3a) $$\begin{array}{l} \alpha =\frac{1-\varepsilon }{2+\varepsilon }\\ \varepsilon =\varepsilon_{0}S_{2}^{2} \end{array},$$

where $\varepsilon_{0}$ is a constant coefficient and,

(3b) $$S_{2}^{2}=\frac{3}{2}\left[\mathrm{tr}\left({\langle uu\rangle }_{\mathrm{an}}\cdot {\langle uu\rangle }_{\mathrm{an}}\right)\right],$$

is the square of the second-order nematic order parameter [23], which depends on the anisotropic orientation tensor ${\langle uu\rangle }_{\mathrm{an}}$ defined as [19,20],

(3c) $${\langle uu\rangle }_{\mathrm{an}}\equiv \langle uu\rangle -\frac{1}{3}I.$$

where **I** is the unit tensor and **u** the unit end-to-end vector of polymer chains. The link tension coefficient controls the anisotropy of the friction tensor as proposed by Curtiss and Bird [15,16,17]. Since, within the Curtiss–Bird theory, the link tension coefficient is associated with the fourth rank orientation tensor, it is natural to allow it to depend on the fourth-order nematic parameter *S*_4_ [23]. To simplify this, we consider the simplest closure approximation, the quadratic one $S_{4}=S_{2}^{2}$ following Stephanou et al. [19,20], thus leading to Equation (3a). Clearly, other expressions could have been used that still nullify the link tension coefficient at equilibrium and allow it to reach a constant value at large strain rates. For example, the ansatz $S_{4}=S_{2}-S_{2}{\left(1-S_{2}\right)}^{\nu }$ with 0<ν<1 has been proposed by Ehrentraut and Hess [24], which boils down to the one we use for *ν* = 1 [23,25]. Herein, we considered the simplest such expression following our previous work [19,20]. Note that the link tension coefficient, as defined in Equation (3a), is not constant: It vanishes at equilibrium, it increases with time in start-up flows, and its steady-state value increases with the imposed strain rate [19]. By considering $\langle uu\rangle =c/\mathrm{tr}c$, then

(3d) $$S_{2}^{2}=\frac{3}{2}\left[\frac{\mathrm{tr}c^{2}}{{\left(\mathrm{tr}c\right)}^{2}}-\frac{1}{3}\right]=1-\frac{3I_{2}}{2I_{1}^{2}}.$$

Note that in the modified Giesekus model, the Giesekus parameter depends on both the first, $I_{1}=\mathrm{tr}c$, and second, $I_{2}=\frac{1}{2}\left[{\left(\mathrm{tr}c\right)}^{2}-\mathrm{tr}c^{2}\right]$, invariants of the conformation tensor, contrary to the Ilg–Kröger (IK) model [21], which exhibits a dependency only on the first invariant (the trace); however, we should emphasize that their revised model is only a simplification of a more general model they have proposed in which, in general, the Giesekus parameter depends also on the second invariant. Since the order parameter asymptotes to unity at large strain rate for a fully aligned sample, the Giesekus parameter approaches the value $\alpha =\left(1-\varepsilon_{0}\right)/\left(2+\varepsilon_{0}\right)$. As a final note, since thermodynamic admissibility dictates that the Giesekus parameter should be 0≤α≤1 [22,26,27,28] and since $0\leq S_{2}\leq 1$ [23,25] and ε≥0 [19,20], we need to bound $0\leq \varepsilon_{0}<1$. Finally, note that when *α* = 0 the Giesekus model simplifies to the upper-convected Maxwell (UCM) model.

It is important to provide a more in-depth comparative discussion regarding the differences between the IK [21] and the modified Giesekus models. Ilg and Kröger [21] proposed a method to develop closed-form and thermodynamically consistent constitutive equations of complex fluids from microscopic models, which were parametrized using thermodynamically guided simulations. This method was exemplified for the case of low-molecular polymer melts subjected to homogeneous flow fields. They proposed a revised expression for the non-equilibrium entropy function, which included, in addition to the known terms of the harmonic dumbbell or Rouse models, a second-order term involving the second invariant of the gyration tensor, namely, the average dyadic of the radius-of-gyration vector of polymer chains. (Note that this tensor cannot be related, in general, to the conformation tensor that we use in this work.) This expression was validated using data accumulated from coarse-grained non-equilibrium molecular dynamics (NEMD) simulations via thermodynamic integration. Then, they showed that the evolution equation for the stress tensor exhibits a second-order term whose coefficient is a function, in general, of all three invariants of the conformation tensor because of the more general non-equilibrium entropy function they considered. Their model was also validated using reference NEMD simulations.

### Asymptotic Behavior of the Model for Steady-State and Start-Up Shear Flow

We then analyze the asymptotic behavior of the two versions of the Giesekus model in the case of a steady and start-up simple shear flow (SSF), described by the kinematics $u=\left(\dot{\gamma }y,0,0\right)$ where *y* denotes the Cartesian coordinate and $\dot{\gamma }$ the shear rate. We present the following material functions: the shear viscosity, $\eta =\sigma_{\mathrm{yx}}/\dot{\gamma }$, and the first and second normal stress coefficients, $\Psi_{1}=\left(\sigma_{\mathrm{xx}}-\sigma_{\mathrm{yy}}\right)/{\dot{\gamma }}^{2}$ and $\Psi_{2}=\left(\sigma_{\mathrm{yy}}-\sigma_{\mathrm{zz}}\right)/{\dot{\gamma }}^{2}$, respectively. The asymptotic behavior is the same as the original Giesekus model, minus the fact that in the modified model close to equilibrium we get *α* = ½, irrespective of the choice of $\varepsilon_{0}$ (see Equation (3)). Thus, for the original Giesekus model,

(4a) $$\begin{array}{l} c_{xx}=1+\left(2-\alpha \right){\mathrm{Wi}}^{2}\\ c_{xy}=\mathrm{Wi}\\ c_{yy}=1-\alpha {\mathrm{Wi}}^{2}\\ c_{zz}=1 \end{array},$$

whereas for the modified Giesekus model,

(4b) $$\begin{array}{l} c_{xx}=1+\frac{3}{2}{\mathrm{Wi}}^{2}\\ c_{xy}=\mathrm{Wi}\\ c_{yy}=1-\frac{1}{2}{\mathrm{Wi}}^{2}\\ c_{zz}=1 \end{array}.$$

In both cases, we have defined the Weissenberg number as $\mathrm{Wi}=\dot{\gamma }\tau_{R}$. We thus obtain the zero-shear-rate material functions for the original model,

(5a) $$\begin{array}{l} \eta_{0}=G\tau_{R}\\ \Psi_{1,0}=2\eta_{0}\tau_{R}\\ -\Psi_{2,0}=\alpha \eta_{0}\tau_{R} \end{array},$$

and for the modified model,

(5b) $$\begin{array}{l} \eta_{0}=G\tau_{R}\\ \Psi_{1,0}=2\eta_{0}\tau_{R}\\ -\Psi_{2,0}=\frac{1}{2}\eta_{0}\tau_{R} \end{array}.$$

During the start-up of simple shear flow, we follow the methodology of Stephanou et al. [28] to obtain the explicit solutions for the time-dependent viscometric functions in the linear viscoelastic (LVE) limit. The results are the same as those of the original Giesekus model,

(6a) $$\begin{array}{l} \eta^{+}(t)=\eta_{0}\left[1-\exp\left(-\frac{t}{\tau_{R}}\right)\right]\\ \Psi_{1}^{+}(t)=\Psi_{1,0}\left[1-\left(1+\frac{t}{\tau_{R}}\right)\exp\left(-\frac{t}{\tau_{R}}\right)\right]\\ \Psi_{2}^{+}(t)=\alpha \eta_{0}\tau_{R}\left[1-\frac{2t}{\tau_{R}}\exp\left(-\frac{t}{\tau_{R}}\right)-\exp\left(-\frac{2t}{\tau_{R}}\right)\right] \end{array},$$

except for the fact that the LVE envelope of the second normal stress coefficient obtained for the modified version differs:

(6b) $$\Psi_{2}^{+}(t)=\frac{1}{2}\eta_{0}\tau_{R}\left[1-\frac{2t}{\tau_{R}}\exp\left(-\frac{t}{\tau_{R}}\right)-\exp\left(-\frac{2t}{\tau_{R}}\right)\right].$$

## 3. Results and Discussion

### 3.1. Model Predictions in Steady-State Shear Flow

**Figure 1:**
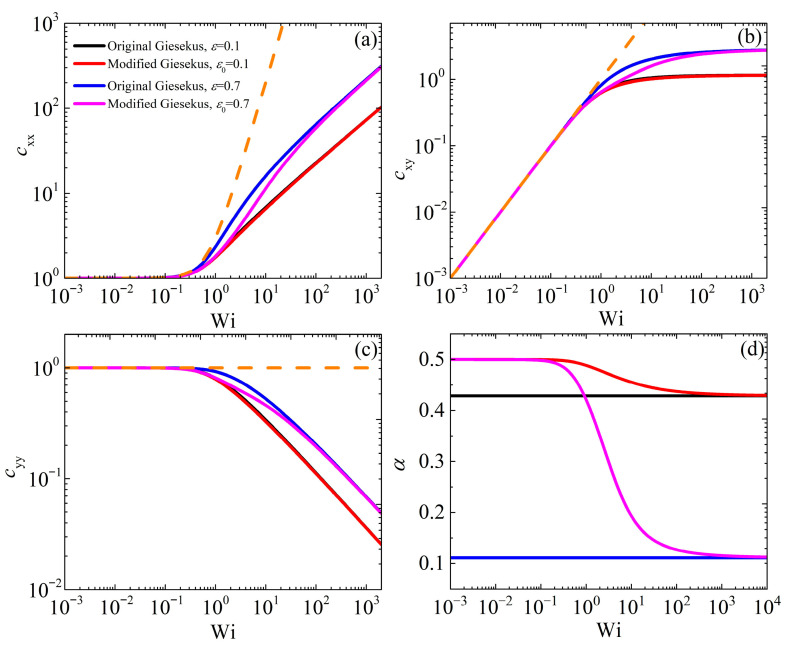
Model predictions for the conformation tensor components (**a**) $c_{xx}$, (**b**) $c_{xy}$, (**c**) $c_{yy}$, and for (**d**) *α* in steady SSF as a function of the dimensionless shear rate and dependency on the parameter $\alpha =\left(1-\varepsilon_{0}\right)/\left(2+\varepsilon_{0}\right)$ for the original model and $\varepsilon_{0}$ for the modified model. The dotted orange lines in panels (**a**–**c**) depict the predictions of the UCM model (with *α* = 0). Note that in panels (**a**–**c**), the red curve almost overlaps the black curve.

**Figure 2:**
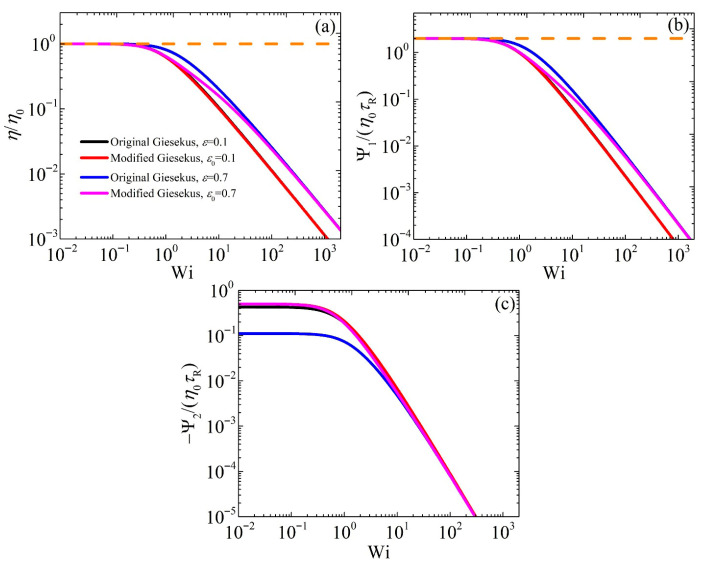
Model predictions for the scaled viscometric functions (**a**) $\bar{\eta }$, (**b**) ${\bar{\Psi }}_{1}$, (**c**) $-{\bar{\Psi }}_{2}$ in steady SSF as a function of the dimensionless shear rate and dependency on the parameter *ε* for the original model and $\varepsilon_{0}$ for the modified model. The dotted orange lines in panels (**a**,**b**) depict the predictions of the UCM model (with *α* = 0). Note that in panels (**a**,**b**), the red curve almost overlaps the black curve, and in panel (**c**), the magenta line almost overlaps both the black and red curves.

Figure 1 illustrates the variations in the conformation tensor components and of *α* as functions of the dimensionless shear rate (Wi) in the case of steady-state SSF when the original Giesekus model is compared with the modified model at two different values of ε or $\varepsilon_{0}$ (0.1 and 0.7); note that these values are close to the domain limits of $\varepsilon_{0}$, $0\leq \varepsilon_{0}<1$. We further mention that when $\varepsilon_{0}=0$, the modified model is identical to the original Giesekus model (or equivalently, the simplified Leonov model [6]) with α=0.5. The behavior of $c_{xx}$ as a function of Wi is the same for both models when small values of ε or $\varepsilon_{0}$ (=0.1) are used. Similarly, as expected for small Wi (Wi < 0.1), $c_{xx}$ remains close to its equilibrium value of unity in both models, following the UCM model predictions (with *α* = 0), and then increases as Wi increases. This is expected since in the linear regime, the mobility tensor equals the unit tensor. However, when ε or $\varepsilon_{0}$ is raised to 0.7, we note that the two models provide different predictions in the intermediate range (approx. 0.1 < Wi < 100), since *α* changes with Wi [Figure 1d], with the modified model consistently predicting lower $c_{xx}$ values than the original model. This is attributed to the large value of the Giesekus parameter relative to the original Giesekus model in this range; larger α values lead to a more anisotropic mobility, related to an increased anisotropy of the orientation of the surrounding polymer chains [6]. However, at large shear rates, the two models agree again, since in this case, $S_{2}\approx 1$ and due to Equation (3a), $\varepsilon \approx \varepsilon_{0}$. A similar behavior is also noted for $c_{xy}$ [Figure 1b] and $c_{yy}$ [Figure 1c]: In both cases, the predictions of the two models are the same at small and large shear rates, whereas the prediction of the modified model is below the original model’s in the intermediate range. The scaling at large shear rates is the same for both models: $c_{xx}$ increases as ${\mathrm{Wi}}^{\frac{1}{2}}$, $c_{xy}$ reaches an asymptotic value, and $c_{yy}$ decreases as ${\mathrm{Wi}}^{-\frac{1}{2}}$. Then, Figure 2 shows the variations of the material viscometric functions as a function of Wi for both the modified and original models. For the shear viscosity and the first normal stress coefficient, we note a similar behavior as for the conformation tensor: The two models provide similar predictions for $\bar{\eta }=\eta /\eta_{0}$ and ${\bar{\Psi }}_{1}=\Psi_{1}/\left(\eta_{0}\tau_{R}\right)$ for small and large shear rates, but the prediction of the modified model is below that of the original model predictions in the intermediate range. Both models predict zero-shear-rate values for both observables in line with experimental data [6,7]. On the contrary, the behavior in the scaled second normal stress coefficient, $-{\bar{\Psi }}_{2}=-\Psi_{2}/\left(\eta_{0}\tau_{R}\right)$, is quite different [Figure 2c]. The two models provide a similar prediction for a small value of ε or $\varepsilon_{0}$ (=0.1), with a similar zero-shear rate value (equal to 9/21 for the original model and ½ for the modified model) as dictated by Equation (5), and the scaling at large shear rates is $\eta \propto {\mathrm{Wi}}^{-1}$, $\Psi_{1}\propto {\mathrm{Wi}}^{-\frac{3}{2}}$, and $-\Psi_{2}\propto {\mathrm{Wi}}^{-2}$ (Table 7.3-5 of Bird et al. [7]); however, they differ significantly at low and intermediate shear rates at larger values of ε or $\varepsilon_{0}$ (=0.7), since the original Giesekus model predicts $-{\bar{\Psi }}_{2}$ to be equal to 1/9, whereas the modified version always predicts it to be equal to ½. This also has implications for the start-up behavior, as will be described below. The power-law slope of the shear viscosity is noted to be unrealistically steep, since experimentally it is found to be between −0.4 and −0.9 [7].

### 3.2. Model Predictions in Steady-State Uniaxial Elongational Flow

In the case of uniaxial elongational flow (UEF), described by the kinematics $u=\left(\dot{\varepsilon }x,-\frac{1}{2}\dot{\varepsilon }y,-\frac{1}{2}\dot{\varepsilon }z\right)$, where $\dot{\varepsilon }$ is the elongation rate (*x* is the flow direction, *y* is the velocity gradient direction, and *z* is the neutral direction), the material function to be analyzed is the elongational viscosity, $\eta_{E}=\left(\sigma_{\mathrm{xx}}-\sigma_{\mathrm{yy}}\right)/\dot{\varepsilon }$. A simple analytical solution can easily be obtained:

(7a) $$\begin{array}{l} c_{xx}=\frac{1}{2\alpha }\left\{\sqrt{1+4{\mathrm{Wi}}^{2}-4\alpha \left(1-2\alpha \right)\mathrm{Wi}}-\left(1-2\alpha -2\mathrm{Wi}\right)\right\}\\ c_{yy}=\frac{1}{2\alpha }\left\{\sqrt{1+{\mathrm{Wi}}^{2}+4\alpha \left(1-2\alpha \right)\mathrm{Wi}}-\left(1-2\alpha +\mathrm{Wi}\right)\right\} \end{array}.$$

so that

(7b) $$\frac{\eta_{E}}{\eta_{0}}=\frac{1}{2\alpha }\left\{3+\frac{1}{\mathrm{Wi}}\sqrt{1+4{\mathrm{Wi}}^{2}-4\alpha \left(1-2\alpha \right)\mathrm{Wi}}-\sqrt{1+{\mathrm{Wi}}^{2}+4\alpha \left(1-2\alpha \right)\mathrm{Wi}}\right\}.$$

Here, $\mathrm{Wi}=\dot{\varepsilon }\tau_{R}$. The resulting Equation (7b) is well known (see Table 7.3-5 with *λ*_2_ = 0 in Bird et al. [7]). In the case of the original Giesekus model, the Giesekus parameter is a constant, whereas in the modified version, it is given from Equations (3a) and (7a), which are semi-analytical since they need to be solved numerically. In both models, the elongational viscosity reaches the asymptote $\eta_{E}/\eta_{0}=2/\alpha$, as we may easily note from Equation (7b) (also see Equation (7B.8-4) of Bird et al. [7]).

**Figure 3:**
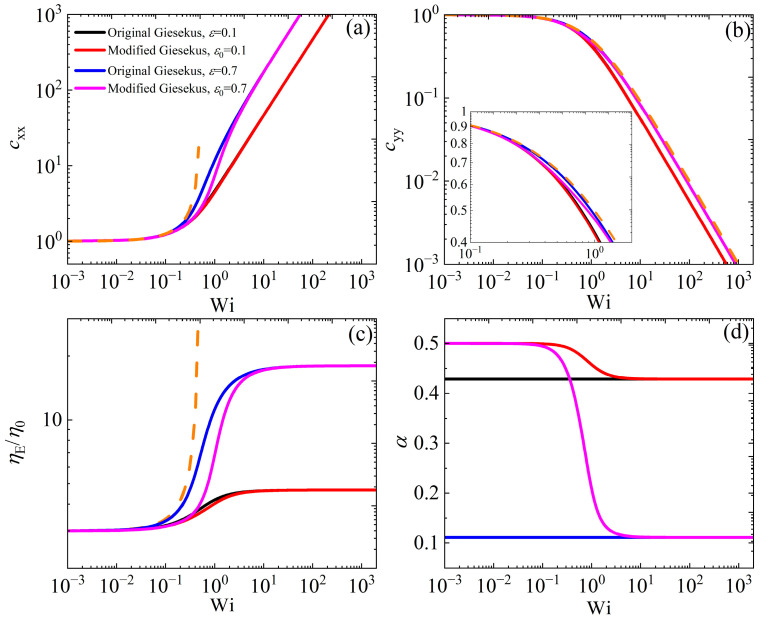
Model predictions for the conformation tensor components (**a**) $c_{xx}$ and (**b**) $c_{yy}$. (**c**) The scaled elongational viscosity ${\bar{\eta }}_{E}=\eta_{E}/\eta_{0}$ and (**d**) *α* in steady UEF as a function of the dimensionless elongation rate and dependency on the parameter *α* for the original model and $\varepsilon_{0}$ for the modified model. The orange dotted lines depict the predictions of the UCM model (with *α* = 0). Note that in panels (**a**–**c**), the red curve almost overlaps the black curve.

Figure 3 shows the conformation tensor elements $c_{xx}$, $c_{yy}$, the dimensionless elongational viscosity (${\bar{\eta }}_{E}\equiv \eta_{E}/\eta_{0}$), and *α* versus Wi. In Figure 3a, $c_{xx}$ starts from its equilibrium value of unity at low Wi for both models, in agreement with the UCM model predictions (with *α* = 0), and then increases with Wi, with higher values as ε or $\varepsilon_{0}$ increases to 0.7. Similarly, in Figure 3b, $c_{yy}$ also starts from its equilibrium value of unity at low Wi for both models, but then decreases as the shear rate increases, exhibiting higher values as ε or $\varepsilon_{0}$ increases. The elongational viscosity starts from the value of 3, as per Trouton’s law, which is in line with experimental data [6,7], and increases as the elongation rate increases, exhibiting smaller values as ε or $\varepsilon_{0}$ increases [Figure 3c]. When $\varepsilon_{0}$= 0.1, both models provide similar predictions for both $c_{xx}$ and $c_{yy}$, whereas the prediction of the modified model is below the prediction of the original model for $c_{xx}$, since *α* is larger in this Wi range [Figure 1d]; on the contrary, it seems that the prediction for $c_{yy}$ is the same for both models, even with a larger value of ε or $\varepsilon_{0}$. The predictions for ${\bar{\eta }}_{E}$ are noted to be the same for both models at large values of ε or $\varepsilon_{0}$, whereas at smaller values, the modified version is below the original Giesekus model’s prediction in the intermediate range of elongation rates. The scaling at large shear rates is the same for both models, namely, ${\tilde{c}}_{xx}\propto \mathrm{Wi}$ and ${\tilde{c}}_{yy}\propto {\mathrm{Wi}}^{-1}$, whereas, as mentioned above, the elongational viscosity always reaches the asymptote ${\bar{\eta }}_{E}=2/\alpha$. This is in contrast to experimental evidence that suggests that the elongational viscosity reaches a maximum value and then decreases at higher elongation rates [7].

### 3.3. Model Predictions in Start-Up Shear Flow

**Figure 4:**
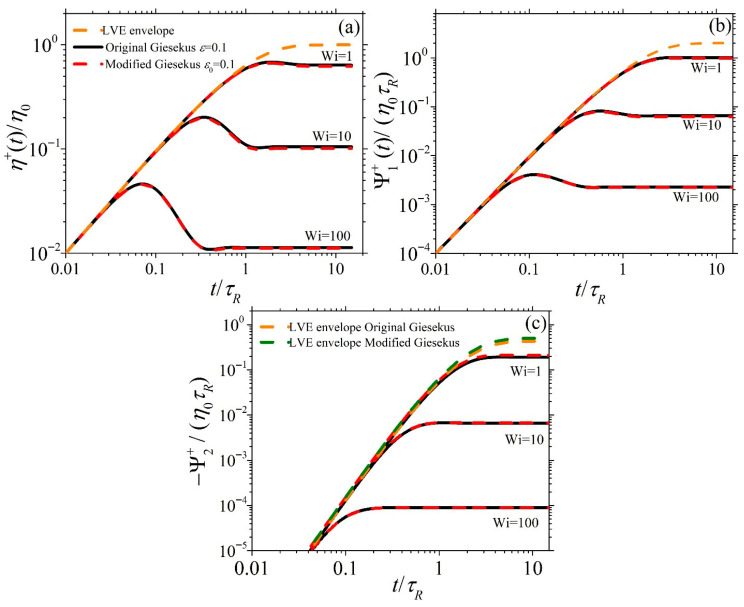
Model predictions when $\varepsilon_{0}=0.1$ for the growth of the (**a**) shear viscosity, (**b**) first normal stress coefficient, and (**c**) second normal stress coefficient, upon the inception of shear flow at different dimensionless shear rates as a function of dimensionless time. The dotted orange lines in each panel and the dotted olive line in panel (**c**) depict the LVE envelope given by Equation (6). Note that the red curve almost overlaps the black curve in all cases, and for this reason, the red curve is dashed.

**Figure 5:**
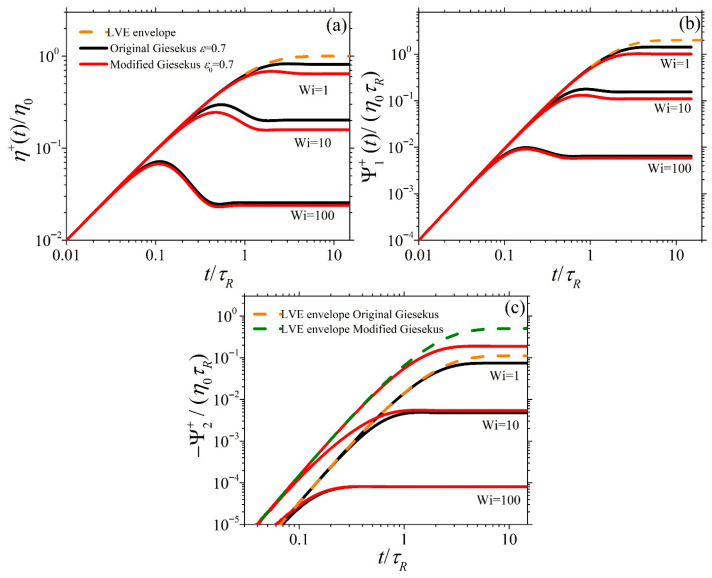
Model predictions when $\varepsilon_{0}=0.7$ for the growth of the (**a**) shear viscosity, (**b**) first normal stress coefficient, and (**c**) second normal stress coefficient, upon the inception of shear flow at different dimensionless shear rates as a function of dimensionless time. The orange dotted lines in each panel and the olive dotted line in panel (**c**) depict the LVE envelope given by Equation (6). Note that the red curve almost overlaps the black curve in many cases.

Figure 4 and Figure 5 show the dimensionless material functions ${\bar{\eta }}^{+}\equiv \eta^{+}/\eta_{0}$, ${\bar{\Psi }}_{1}^{+}\equiv \Psi_{1}^{+}/\left(\eta_{0}\tau_{R}\right)$, and $-{\bar{\Psi }}_{2}^{+}\equiv -\Psi_{2}^{+}/\left(\eta_{0}\tau_{R}\right)$ for start-up SSF as a function of dimensionless time for $\varepsilon_{0}$ = 0.1 and $\varepsilon_{0}$ = 0.7, respectively, at Wi = 1, 10, and 100. In Figure 4a,b, the predictions of ${\bar{\eta }}^{+}$ and ${\bar{\Psi }}_{1}^{+}$ for both models exhibit similar overall trends for all Wi: They increase at early times following the LVE envelope (Equation (6)), reach an overshoot, and then decrease before reaching their steady state value; this is in line with experimental evidence [6,7]. Also, both models’ shear viscosity predictions present an undershoot after the overshoot, which is more easily noticeable at Wi = 100. This oscillatory behavior for the original Giesekus model was first noted by Giesekus [13]. Such undershoots, first provided by the experimental work of Costanzo et al. [29], have been associated with the tumbling of polymer chains in simple shear [30,31]. When ε or $\varepsilon_{0}$ is small (=0.1), the predictions of the original and modified Giesekus models are identical, as seen in Figure 4, whereas at a larger value (=0.7), there are clear differences between the original and modified models (Figure 5). In particular, the predictions of the modified Giesekus model for ${\bar{\eta }}^{+}$ and ${\bar{\Psi }}_{1}^{+}$ are noted to be below the original Giesekus model predictions at large times for intermediate shear rates (Wi = 1 and 10), whereas the predictions are similar at larger shear rates irrespective of time. On the other hand, the predictions of the two models for $-{\bar{\Psi }}_{2}^{+}$ are noted to be completely different even in the LVE regime, as indicated by the different expressions for the LVE envelope [see Equation (6)]. The modified model is seen to shift upwards following Equation (6b) and is noted to predict a larger value than the original model at small shear rates (Wi = 1). However, at larger shear rates (Wi = 10 and 100), both models exhibit the same steady-state value. This behavior is more in line with experimental data [19]; as seen in Figure 8b of Stephanou et al. [28], the LVE envelope predicted by the original Giesekus model (note that the LVE envelope provided there is not the same as the original Giesekus model provided here; however, due to the small value of the slip parameter considered in their work, *ξ* = 0.03, their LVE envelope should not differ substantially from the original Giesekus model one) is below the LVE envelope suggested by the experimental data. Thus, the use of a variable Giesekus parameter, as done in the modified model, would certainly improve the comparison against said experimental data.

### 3.4. Comparison Against the Leonov and IK Models

**Figure 6:**
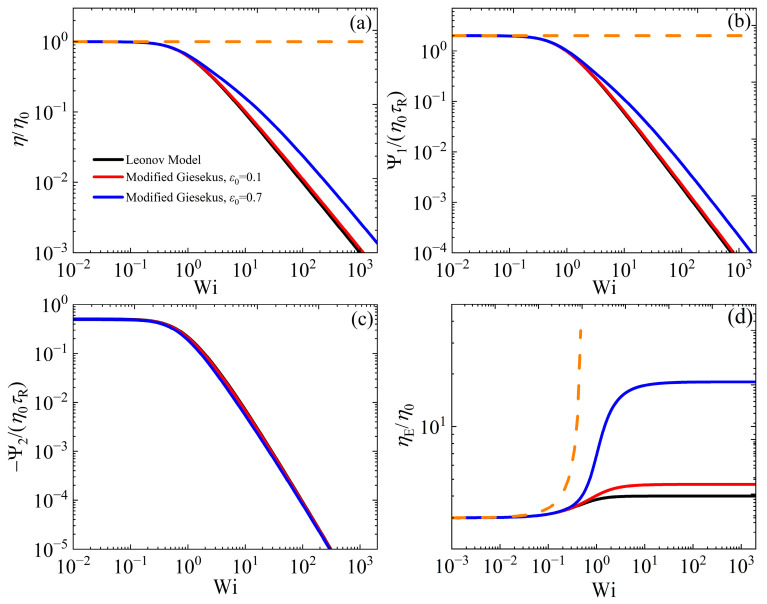
Model predictions for the dimensionless viscometric functions (**a**) shear viscosity, (**b**) first and (**c**) second normal stress coefficient, in steady SSF, as a function of the dimensionless shear rate, and (**d**) the scaled elongational viscosity $\eta_{E}$ in steady UEF as a function of the dimensionless elongation rate, for the Leonov and the modified Giesekus model. The orange dotted lines depict the predictions of the UCM model (with α = 0). Note that in panels (**a**,**b**), the red curve almost overlaps the black curve, and in panel (**c**), the blue line almost overlaps both the black and red curves.

In Figure 6a,c, we illustrate the variations in the viscometric functions, as functions of the dimensionless shear rate (Wi), in the case of steady-state SSF when the Leonov model is compared with the modified Giesekus model at two different values of $\varepsilon_{0}$ (0.1 and 0.7). Similarly, Figure 6d illustrates the scaled elongational viscosity, $\eta_{E}/\eta_{0}$, in steady-state UEF, in which case the Leonov model is analytically solvable; the solution is the same as Equation (7) with *α* = 0.5. (The corresponding variations in the conformation tensor components are provided in Figure S1 for SSF and Figure S2 for UEF, respectively.) We note that when $\varepsilon_{0}$ = 0.1, the modified Giesekus model predictions are almost the same as the Leonov model’s ones, which is expected since the Giesekus parameter in the modified model changes only slightly (from 0.5 at equilibrium to $\alpha =\left(1-\varepsilon_{0}\right)/\left(2+\varepsilon_{0}\right)$ = 3/7 ≈ 0.43 at Wi >> 1). However, when $\varepsilon_{0}$ = 0.7, the predictions of the modified Giesekus model are above Leonov’s in the non-linear regime as expected, since the Giesekus parameter in the modified model changes considerably (from 0.5 at equilibrium to 1/9 ≈ 0.11 at Wi >> 1). The only exception is the second normal stress coefficient [Figure 6c], which is seen to be almost the same as in the Leonov model. The corresponding comparison in start-up shear flow is provided in Figures S3 and S4.

The evolution equation for the dimensionless gyration tensor, **G**, of the IK model is given as

(8a) $${\dot{G}}_{\left[1\right]}=-\frac{1}{\tau_{R}}\left\{\alpha \theta \left(\mathrm{tr}G\right)G\cdot G-\left[\left(1-\alpha \right)\theta \left(\mathrm{tr}G\right)-\alpha \right]G+\left(1-\alpha \right)I\right\},$$

where $\theta \left(\mathrm{tr}G\right)\equiv 1+\frac{4}{9}\chi \left(\mathrm{tr}G-3\right)$ and we have considered $2\nu =\left(1-\alpha \right)/\tau_{R},\mu =\alpha /\tau_{R}$ since these two parameters are not independent, since at equilibrium the dimensionless mobility tensor should coincide with the unit tensor. The corresponding equation for the extra stress tensor is

(8b) $$\sigma =G_{0}\left\{\theta \left(\mathrm{tr}G\right)G-I\right\}.$$

**Figure 7:**
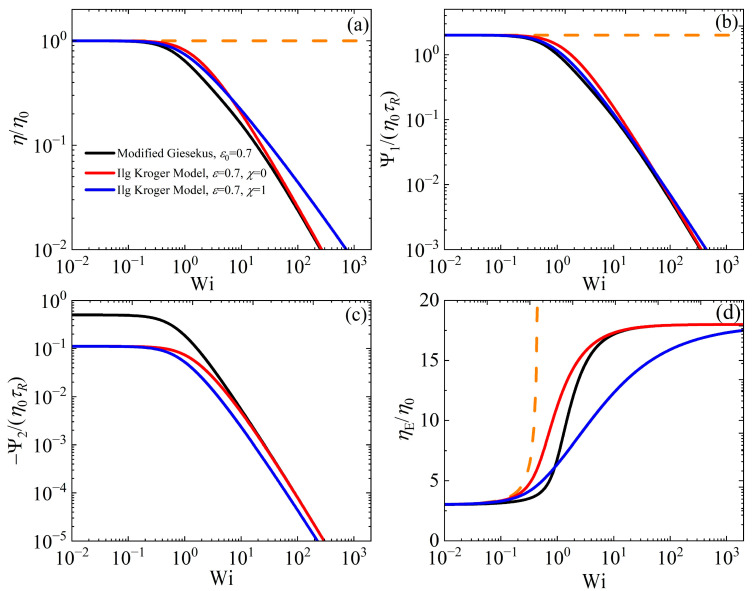
Model predictions for the dimensionless viscometric functions (**a**) shear viscosity, (**b**) first and (**c**) second normal stress coefficient, in steady SSF, as a function of the dimensionless shear rate, and (**d**) the scaled elongational viscosity $\eta_{E}$ in steady UEF as a function of the dimensionless elongation rate, for the IK model, with ε = 0.7 (α = 1/9) and *χ* = 0 and 1, and the modified Giesekus model with $\varepsilon_{0}$ = 0.7. The orange dotted lines depict the predictions of the UCM model (with α = 0). Note that in panel (**b**), the blue curve almost overlaps the black curve.

The IK model, as presented here, includes two parameters: *α* and *χ*. When *χ* = 0, it coincides with the original Giesekus model. The variations in the viscometric functions, as functions of the dimensionless shear rate (Wi) in the case of steady-state SSF, when the IK model is compared with the modified model with $\varepsilon_{0}$ = 0.7, meaning $\alpha =\left(1-\varepsilon_{0}\right)/\left(2+\varepsilon_{0}\right)$ = 1/9 ≈ 0.11 at Wi >> 1, which is the value selected in the IK model, are presented in Figure 7a–c, whereas Figure 7d illustrates $\eta_{E}/\eta_{0}$ in steady-state UEF. Note that since the structural variable is not the same in the two models (conformation tensor based on the chain end-to-end vector in our case, and gyration tensor in the IK model), it is not possible to compare the predictions between the two structural variables. Also, since *α* is a common parameter in both models (as we chose to present the IK model here), we only need to describe the influence of *χ* on the various material functions. The modified Giesekus model is noted to decrease faster at intermediate Wi values (approx. 0.5 < Wi < 10), but as the *χ* value in the IK model increases, the difference between the two models becomes smaller. Furthermore, at large shear rates, by increasing *χ*, the IK model exhibits a smaller power-law behavior, approx. −0.77, which is noted to be more in accord with experimental data. On the other hand, ${\bar{\Psi }}_{1}$ predictions are noted not to differ significantly (both models exhibit a power-law behavior at large shear rates close to −3/2), whereas the prediction of the IK model for $-{\bar{\Psi }}_{2}$, by increasing *χ* shifts to smaller Wi values, provides smaller values relative to the modified Giesekus model prediction, whereas the power-law behavior at large shear rates is the same. Clearly, as expected, the zero-rate prediction differs. Lastly, we note that increasing *χ* in UEF results in a slower approach to the asymptotic limit at large elongation rates, but this limit is the same for two models. Note that for a smaller $\varepsilon_{0}$ value, the differences between the two models are smaller (cf. Figure S5). The corresponding comparison in start-up shear flow is provided in Figures S6 and S7.

### 3.5. Comparison with Experimental Data

**Figure 8:**
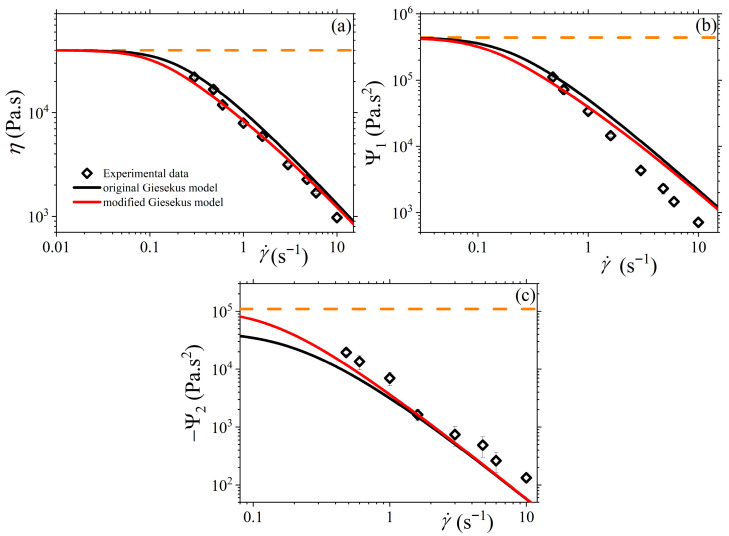
Comparison of the predictions of both the original and modified Giesekus models with the experimental data presented in Ref. [19] for (**a**) the shear viscosity, (**b**) the first normal stress coefficient, and (**c**) the second normal stress coefficient, at steady state. Parameter values: $\eta_{0}=40\;\mathrm{kPa}.s,\tau_{R}=5.5\;s$, and $\varepsilon_{0}=0.5$, or α = 0.2 for the original Giesekus model. The orange dotted lines depict the predictions of the UCM model (with α = 0).

Finally, in Figure 8, we compare the original and modified Giesekus models with the experimental data for the viscometric functions in the case of the steady-state simple shear flow of Stephanou et al. [19]. We select the zero-rate viscosity and the relaxation time as $\eta_{0}=40\;\mathrm{kPa}.s,\tau_{R}=5.5\;s$, and $\varepsilon_{0}=0.5$, or α = 0.2 for the original Giesekus model. Note that the zero-rate first and second normal stress coefficients are given in Equation (5). We note that the modified Giesekus model can more accurately describe the experimental results, particularly of the shear viscosity and the second normal stress coefficient depicted in Figure 6a and Figure 6c, respectively. The modified Giesekus model is also noted to be favorable compared with the first normal stress coefficient data at small shear rates, although it fails to predict it at large shear rates. Overall, the modified model, even without the consideration of additional molecular mechanisms [22], can quantitatively capture the steady-state SSF experimental data of Stephanou et al. [19].

## 4. Conclusions

Giesekus [18], to validate his postulate, showed that his expression for the mobility tensor can be obtained through a linearization of the mobility tensor proposed by Curtiss and Bird [15,16,17], which allowed him to relate his anisotropicity parameter to the link tension coefficient. However, recent evidence by Stephanou et al. [19,20] unambiguously shows that the link tension coefficient should not be considered a constant but should depend on strain rate and time via the nematic order parameter. In this work, we provide the predictions of a modified Giesekus parameter where the Giesekus parameter is no longer constant. Under steady-state simple shear flow, we note that the two models exhibit the same behavior when ε or $\varepsilon_{0}$ is small, and at any value of ε or $\varepsilon_{0}$ at small and large shear rates. A similar behavior is noted for the transient shear viscosity and the transient first normal stress coefficient in start-up simple shear flow; however, the predictions are noted to be very different in the case of the transient second normal stress coefficient in start-up simple shear flow, where the LVE envelope is seen to shift to larger values, which is more in line with experimental data [19,28]. We thus expect that the use of a variable Giesekus parameter will improve the predictive capacity of constitutive models that employ the Giesekus parameter to more accurately predict the rheological behavior of polymeric systems.

We should, however, also discuss some of the limitations of the modified Giesekus model. First, we need to add additional molecular mechanisms such as finite chain extensibility, non-affine deformation, and variation of the longest chain relaxation time with chain conformation [22], which will provide a more robust predictive capacity to the constitutive model. Second, we have herein assumed monodisperse samples, which are never experienced in industrial applications; as such, we should, in the future, further consider polydispersity effects. Furthermore, we have assumed isothermal and incompressible flows, which are seldom used in industry. We will first need to generalize the constitutive model by accounting for the temperature field using non-equilibrium thermodynamics [26] and then employ computational fluid dynamics software to solve it in actual industrial processes, e.g., extrusion. We aim to undertake such improvements in the future.

## Data Availability

The data that support the findings of this study are available within the article.

## Supplementary Materials

The following supporting information can be downloaded at: https://www.mdpi.com/article/10.3390/polym17182510/s1. Figure S1: Model predictions for the conformation tensor components (a) $c_{xx}$, (b) $c_{xy}$, (c) $c_{yy}$, in steady SSF as a function of the dimensionless shear rate Wi, for the Leonov and the modified Giesekus model. The dotted lines depict the predictions of the Upper Convected Maxwell (UCM) model (with α=0); Figure S2: Model predictions for the conformation tensor components (a) $c_{xx}$, and (b) $c_{yy}$, in steady UEF as a function of the dimensionless elongation rate Wi, for the Leonov and the modified Giesekus model. The dotted lines depict the predictions of the UCM model (with α=0); Figure S3: Model predictions for the growth of the (a) shear viscosity, (b) first normal stress coefficient, and (c) second normal stress coefficient, upon the inception of shear flow at different dimensionless shear rates as a function of dimensionless time, for the Leonov and the modified Giesekus model with $\varepsilon_{0}=\;0.1$. The dotted lines in each panel depict the LVE envelope given by Equation (6) of the main text; Figure S4: Model predictions for the growth of the (a) shear viscosity, (b) first normal stress coefficient, and (c) second normal stress coefficient, upon the inception of shear flow at different dimensionless shear rates as a function of dimensionless time, for the Leonov and the modified Giesekus model with $\varepsilon_{0}=\;0.7$. The dotted lines in each panel depict the LVE envelope given by Equation (6) of the main text; Figure S5: Model predictions for the dimensionless viscometric functions (a) shear viscosity, (b) first and (c) second normal stress coefficient, in steady SSF, as a function of the dimensionless shear rate, and (d) the scaled elongational viscosity $\eta_{E}$ in steady UEF as a function of the dimensionless elongation rate, for the IK, with α=3/7 and χ = 0 and 1, and the modified Giesekus model with $\varepsilon_{0}=\;0.1$. The dotted lines depict the predictions of the UCM model (with α=0); Figure S6: Model predictions for the growth of the (a) shear viscosity, (b) first normal stress coefficient, and (c) second normal stress coefficient, upon the inception of shear flow at different dimensionless shear rates as a function of dimensionless time, for the modified Giesekus model with $\varepsilon_{0}=\;0.1$ and the IK model when χ = 0 and 1. The dotted dark yellow lines in each panel and the dotted olive line in panel (c) depict the LVE envelope given by Equation (6); Figure S7: Model predictions for the growth of the (a) shear viscosity, (b) first normal stress coefficient, and (c) second normal stress coefficient, upon the inception of shear flow at different dimensionless shear rates as a function of dimensionless time, for the IM model and the modified Giesekus model with $\varepsilon_{0}=\;0.7$. The dotted dark yellow lines in each panel and the dotted olive line in panel (c) depict the LVE envelope given by Equation (6).
