# Supplementary material for: Considering a Non-Constant Anisotropicity Parameter in the Giesekus Model
Source: Polymers (Basel). 2025 Sep 17;17(18):2510. doi: 10.3390/polym17182510 (PMC12473250; doi:10.3390/polym17182510)
Supplement: Supplementary file 1 [file polymers-17-02510-s001.zip › polymers-3799605-supplementary.pdf]

SUPPLEMENTARY MATERIAL
Considering a non-constant anisotropicity parameter in the Giesekus model

Fatemeh Karami[1] and Pavlos S. Stephanou[2,*]
[1]*Department of Mechanical Engineering, Lorestan University, Khorramabad, Iran*
[2]*Department of Chemical Engineering, Cyprus University of Technology, PO Box 50329, 3603 Limassol, Cyprus*
*Corresponding author, Tel.: +357-25-002394, fax: +357-25-002668, e-mail: pavlos.stefanou@cut.ac.cy

In this Supplementary Material (SM), we provide additional comparison, relative to the one presented in the main text, between the modified Giesekus model and the simplified Leonov model (which coincides with the original Giesekus model when α=0.5) [1,2] in Section S1, and the Ilg-Kröger model [3] in Section S2.

S1. Comparison with the Leonov Model

S1.1. Model Predictions in Steady-State Simple Shear Flow (SSF) and Uniaxial Elongational Flow (UEF)

Figure S1 illustrates the variations in the conformation tensor components as functions of the

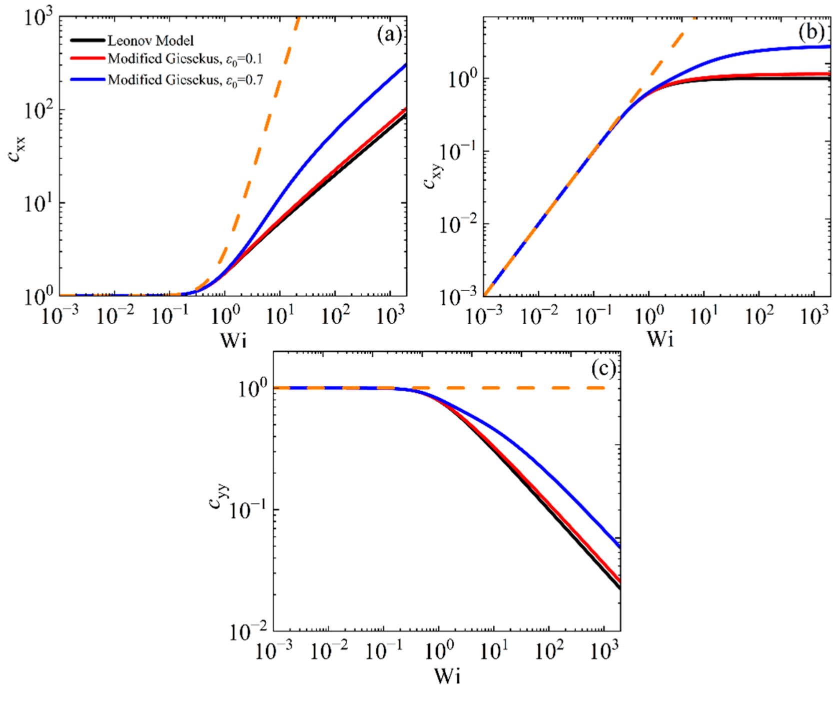

Figure S1. Model predictions for the conformation tensor components (a) c_{xx}, (b) c_{xy}, (c) c_{yy}, in steady SSF as a function of the dimensionless shear rate Wi, for the Leonov and the modified

Giesekus model. The dotted lines depict the predictions of the Upper Convected Maxwell (UCM) model (with α=0).

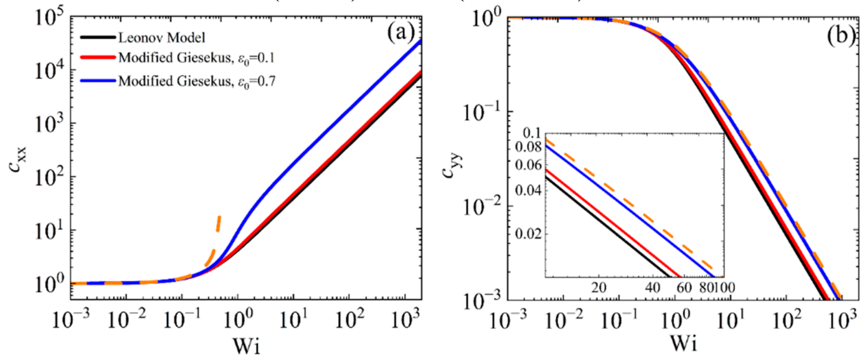

Figure S2. Model predictions for the conformation tensor components (a) c_{xx}, and (b) c_{yy}, in steady UEF as a function of the dimensionless elongation rate Wi, for the Leonov and the modified Giesekus model. The dotted lines depict the predictions of the UCM model (with α=0).

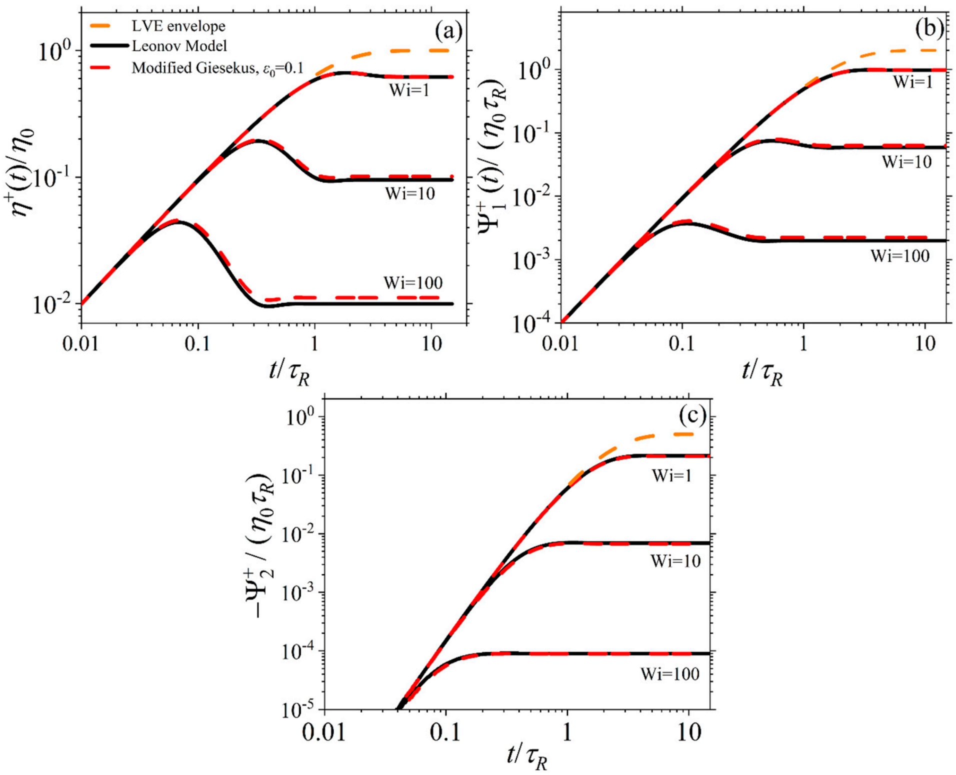

Figure S3. Model predictions for the growth of the (a) shear viscosity, (b) first normal stress coefficient, and (c) second normal stress coefficient, upon the inception of shear flow at different dimensionless shear rates as a function of dimensionless time, for the Leonov and the modified Giesekus model with ε_0 = 0.1. The dotted lines in each panel depict the LVE envelope given by Eq. (6) of the main text.

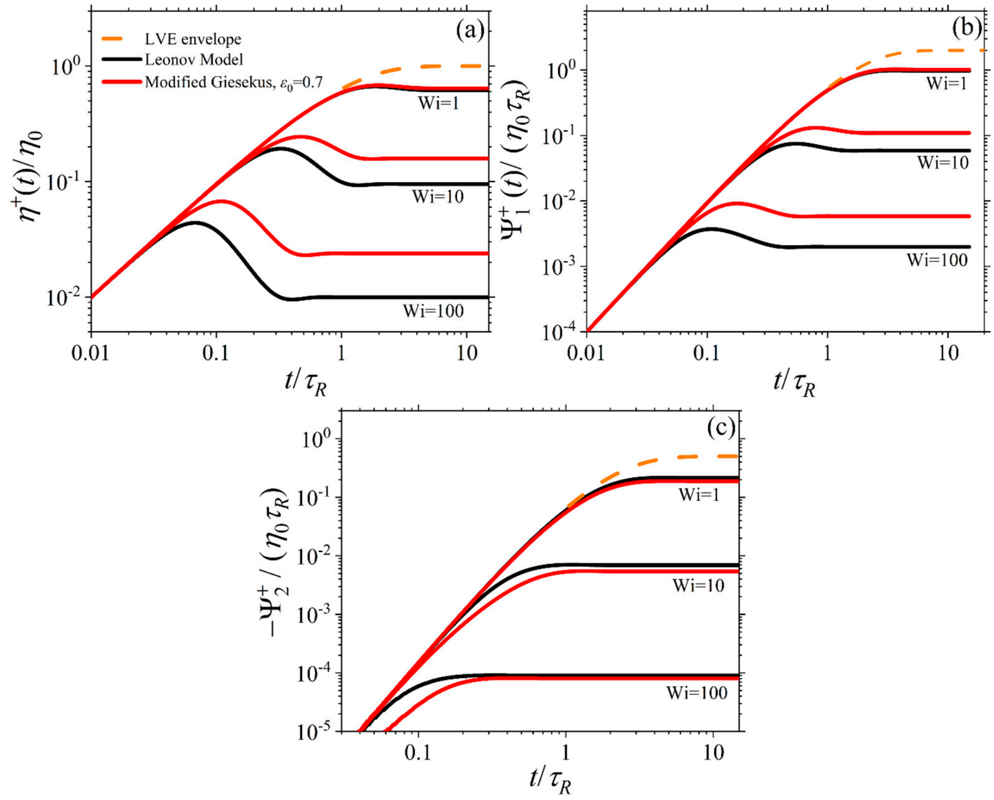

Figure S4. Model predictions for the growth of the (a) shear viscosity, (b) first normal stress coefficient, and (c) second normal stress coefficient, upon the inception of shear flow at different dimensionless shear rates as a function of dimensionless time, for the Leonov and the modified Giesekus model with $\varepsilon_0 = 0.7$. The dotted lines in each panel depict the LVE envelope given by Eq. (6) of the main text.

dimensionless shear rate (Wi) in the case of steady-state SSF when the Leonov model is compared with the modified model at two different values of ε_0 (0.1 and 0.7). Similarly, Figure S2 illustrates the components of the conformation tensor, c_{xx} and c_{yy}, in steady UEF. Similarly to Figure 6, in all
three figures, we note that when ε_0=0.1, the modified Giesekus model predictions are almost the same as the Leonov model's ones whereas, when ε_0=0.7, the predictions of the modified Giesekus model are above Leonov's one in the non-linear regime.

S1.2. Model Predictions in start-up Shear Flow

Figures S3 and S4 show a comparison between the dimensionless material functions η^+/η_0, $\Psi_1^+/(\eta_0\tau_R)$, and $-\Psi_2^+/(\eta_0\tau_R)$ for startup SSF as a function of dimensionless time of the Leonov model and the modified Giesekus model with $\varepsilon_0 = 0.1$ and $\varepsilon_0 = 0.7$, respectively. We again note,

as in the steady-state predictions presented before, that the modified Giesekus model with $\varepsilon_0 = 0.1$ bears very similar predictions to the Leonov model. On the contrary, the predictions of the

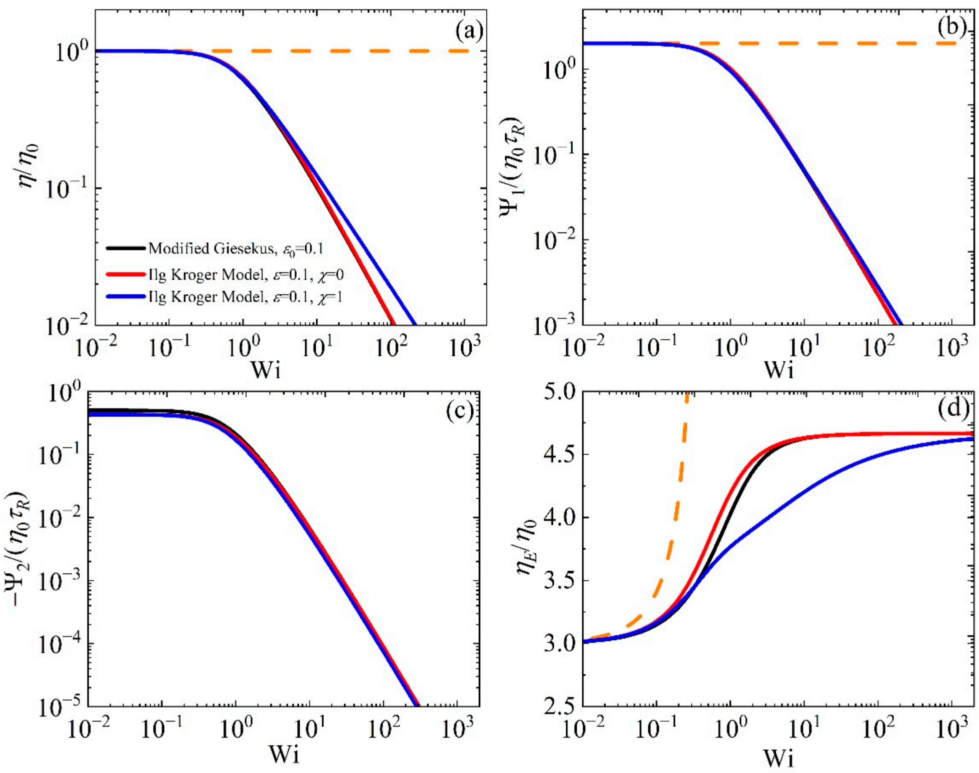

Figure S5. Model predictions for the dimensionless viscometric functions (a) shear viscosity, (b) first and (c) second normal stress coefficient, in steady SSF, as a function of the dimensionless shear rate, and (d) the scaled elongational viscosity η_E in steady UEF as a function of the dimensionless elongation rate, for the IK, with α=3/7 and χ=0 and 1, and the modified Giesekus model with ε_0=0.1. The dotted lines depict the predictions of the UCM model (with α=0).

modified Giesekus model with $\varepsilon_0 = 0.7$ are always above Leonov's ones in the non-linear regime, which intensifies as the Wi increases, except for $-\Psi_2^+/(\eta_0\tau_R)$. Both are expected, given the steady-state predictions provided in Figure 6.

S2. Comparison with the Ilg-Kröger (IK) Model

S2.1. Model Predictions in Steady-State SSF and UEF

In Figure S5(a) to S5(c), we present the variations in the viscometric functions, as functions of the dimensionless shear rate (Wi) in the case of steady-state SSF, when the IK model is compared with the modified model when ε_0=0.1, meaning $\alpha = (1-\varepsilon_0)/(2+\varepsilon_0)$=3/7≈0.43 at Wi>>1, which is the value selected in the IK model, whereas in panel (d), we present the elongational viscosity in steady-state UEF. As mentioned in the main text, by considering a smaller value for ε_0 the differences between the two models become smaller (cf. Figure 7 where ε_0=0.7).

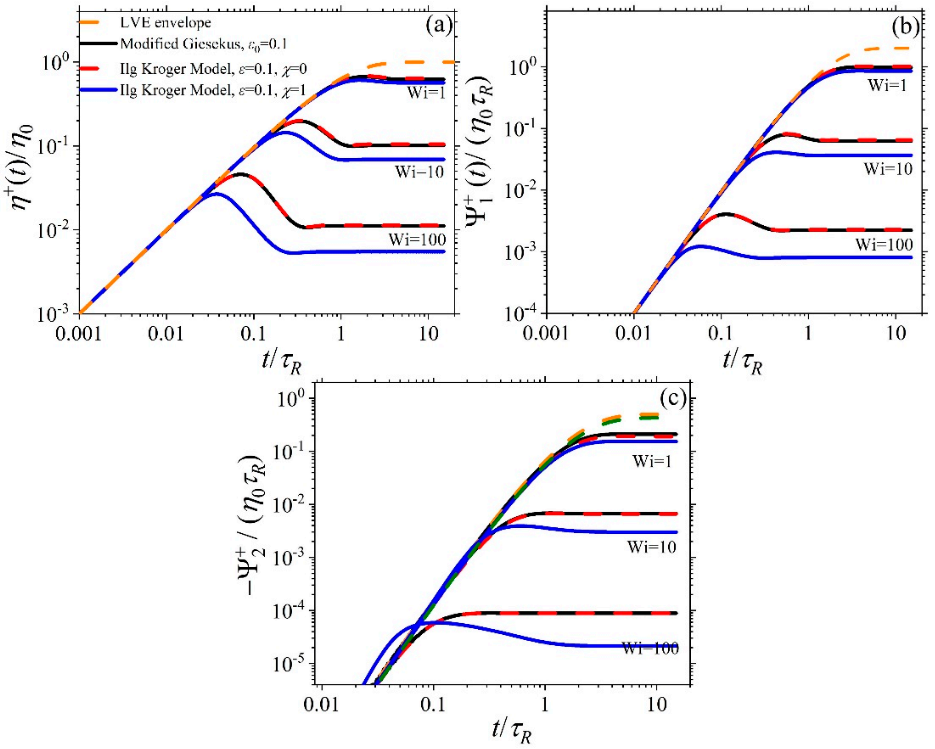

Figure S6. Model predictions for the growth of the (a) shear viscosity, (b) first normal stress coefficient, and (c) second normal stress coefficient, upon the inception of shear flow at different dimensionless shear rates as a function of dimensionless time, for the modified Giesekus model with ε_0 = 0.1 and the IK model when χ=0 and 1. The dotted dark yellow lines in each panel and the dotted olive line in panel (c) depict the LVE envelope given by Eq. (6).

S2.3. Model Predictions in start-up Shear Flow

Figures S6 and S7 show a comparison between the dimensionless material functions η^+/η_0, $\Psi_1^+/(\eta_0\tau_R)$, and $-\Psi_2^+/(\eta_0\tau_R)$ for startup SSF as a function of dimensionless time of the IK model and the modified Giesekus model with ε_0 = 0.1 and ε_0 = 0.7, respectively, and for χ=0 and 1 in the case of the IK model. We note that when χ=0, the two models provide similar predictions, although the predictions when ε_0 = 0.7 (meaning smaller α for the IK model) is noted to be slightly above the modified Giesekus predictions when Wi=1 and 10, whereas when χ=1, the overshoot of both η^+/η_0 and $\Psi_1^+/(\eta_0\tau_R)$ shifts to smaller times and the steady state value reduces, in line with the predictions presented in Figure 7 and Figure S5. A similar comparison is noted for $-\Psi_2^+/(\eta_0\tau_R)$ although the most important difference is the fact that when χ=1, the IK model predicts that at large Wi (noted particularly when Wi=100) are noted to go over the LVE envelope instead of below.

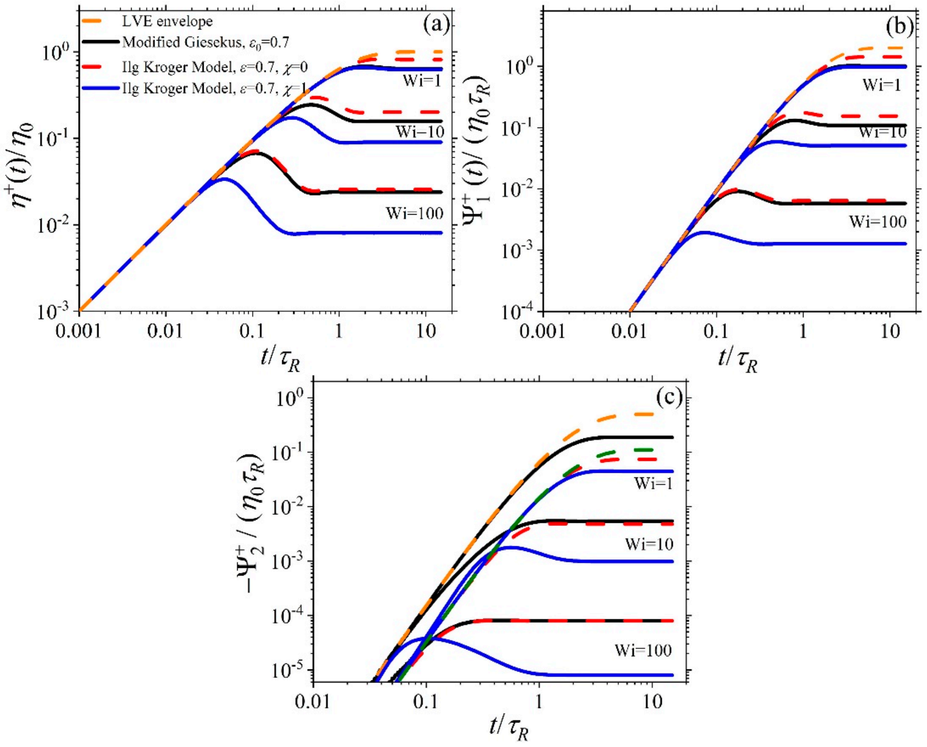

Figure S7. Model predictions for the growth of the (a) shear viscosity, (b) first normal stress coefficient, and (c) second normal stress coefficient, upon the inception of shear flow at different dimensionless shear rates as a function of dimensionless time, for the IM model and the modified Giesekus model with $\varepsilon_0 = 0.7$. The dotted dark yellow lines in each panel and the dotted olive line in panel (c) depict the LVE envelope given by Eq. (6).

References

1. Bird, R.B.; Armstrong, R.C.; Hassager, O. *Dynamics of Polymeric Liquids. Volume 1. Fluid Mechanics.*; 2nd Editio.; Wiley-Interscience, 1987; ISBN 047107375X.
2. Larson, R.G. *Constitutive Equations for Polymer Melts and Solutions*; 1st ed.; Butterworth-Heinemann, 1988; ISBN 978-0-409-90119-1.
3. Ilg, P.; Kröger, M.; Molecularly Derived Constitutive Equation for Low-Molecular Polymer Melts from Thermodynamically Guided Simulation. *J. Rheol.* **2011**, *55*, 69–93, doi:10.1122/1.3523485.
